# Data of the Swiss common breeding bird monitoring program

**DOI:** 10.1002/ecy.70268

**Published:** 2025-12-08

**Authors:** Nicolas Strebel, Samuel Wechsler, Roman Bühler, Guido Häfliger, Verena Keller, Marc Kéry, Christian Rogenmoser, Martin Spiess, Katarina Varga, Bernard Volet, Niklaus Zbinden, Hans Schmid

**Affiliations:** ^1^ Swiss Ornithological Institute Sempach Switzerland

**Keywords:** abundance, annual survey, bird census, breeding season, citizen science, common breeding bird monitoring, long‐term monitoring, structured monitoring, Switzerland, territory mapping

## Abstract

The Swiss Common Breeding Bird Monitoring (“Monitoring Häufige Brutvögel” MHB) is a long‐term study organized by the Swiss Ornithological Institute. Its main goal is to collect data for estimating breeding population trends of relatively abundant and widespread species. Since 1999, 267 one‐km squares laid out in a mostly systematic grid across all of Switzerland have been surveyed annually by skilled, mostly volunteer ornithologists. The sampling sites thus cover a wide range of typical Western European habitats, and an altitudinal range from 250 up to 2750 m above sea level. Bird populations are recorded using a simplified territory mapping protocol with two visits per square above the timberline and three elsewhere. Surveys are conducted during the breeding season (mid‐April to early July) along a square‐specific transect route that does not change over the years. A typical transect route is between 4 and 6 km long, and each visit usually lasts 3 to 4 h. The location of all visually or acoustically detected birds is recorded on topographical maps or using a smartphone app. Records that meet predefined criteria in terms of species‐specific breeding period and observed behavior are retained for the subsequent step of territory delimitation. This is done automatically for most species by the program Autoterri since 2022 and was done manually before, with subsequent checks by an expert. This process finally results in an estimate of the total number of detected territories per species, square and year. The design also explicitly generates detection histories, consisting of two to three numbers that represent the number of territories found to be occupied during each respective visit, enabling the analysis with binomial N‐mixture and site‐occupancy models. The dataset currently covers the breeding seasons from 1999 to 2024 and includes 6852 site‐by‐year combinations with estimates of detected territory numbers. It covers 162 of the 166 bird species recorded at least once as potential breeders, excluding four species to prevent potential disturbance at nesting sites. Besides informing about population trends, data from the Swiss Common Breeding Bird Monitoring were used to illustrate several methodological developments in N‐mixture, occupancy and related models and to answer scientific and applied questions. With its clearly defined survey method, the largely systematic distribution of its survey sites, and the long timespan covered, it is likely that this dataset will continue to make important contributions in biological and biostatistical research. Herewith, we make the annually updated data set available with a CC BY 4.0 license, allowing researchers and conservationists to use and analyze the data for their own research and conservation efforts.

## CONFLICT OF INTEREST STATEMENT

The authors declare no conflicts of interest.

## Supporting information


Data S1.

## Data Availability

The data set is available as Supporting Information and is additionally available in the vogelwarte.ch Open Repository and Archive, hosted on Zenodo, at https://doi.org/10.5281/zenodo.15854964. For the latest annual update, please see https://doi.org/10.5281/zenodo.15854963.

