# Supplementary material for: Data of the Swiss common breeding bird monitoring program
Source: Ecology. 2025 Dec 8;106(12):e70268. doi: 10.1002/ecy.70268 (PMC12683608; doi:10.1002/ecy.70268)
Supplement: Supplementary file 1 — Data S1. [file ECY-106-e70268-s001.zip › Data_S1/Metadata_S1.pdf]

Data Paper published in *Ecology*, an Ecological Society of America journal

Metadata S1

Data of the Swiss Common Breeding Bird Monitoring Program

Nicolas Strebel, Samuel Wechsler, Roman Bühler, Guido Häfliger, Verena Keller, Marc Kéry, Christian Rogenmoser, Martin Spiess, Katarina Varga, Bernard Volet, Niklaus Zbinden, Hans Schmid

Introduction

Monitoring population trends of breeding birds has a long tradition in many countries (Baillie, 1991; PECBMS, 2023; Sauer et al., 2013; Schmeller et al., 2012; van Turnhout et al., 2010). Bird population monitoring provides the basis for effective bird conservation (Jiguet et al., 2013). Furthermore, birds are considered particularly suitable bioindicators, as changes in their population can provide insights into changes in the ecosystems they inhabit (Fraixedas et al., 2020; Gregory et al., 2005). Knowledge of avian population trends allows, for example, quantifying the impact of land use changes on bird populations (Järvinen & Väisänen, 1979). Population trends of bird species also serve as a database for indicators for policy advice (European Environment Agency, 2021; Sudfeldt et al., 2012). Bird monitoring often involves annual surveys of a set of points, plots, or transects (Bibby et al., 2000; PECBMS, 2023). Typically, sampling plots are visited by experienced field ornithologists to determine the occurrence or abundance of individual breeding bird species. In Switzerland, the Swiss Common Breeding Bird Monitoring ("Monitoring Häufige Brutvögel" MHB) was initiated in the late 1990s. Since 1999, a set of 267 one-kilometre squares (=sites) is annually surveyed by well-trained observers (Schmid et al., 2004). For the most part, the observers are volunteers. Visits take place during the breeding season (mid-April to early July overall, but for any given square usually within a period of four to six weeks). Data is gathered by simplified territory mapping, where instead of six to twelve repeat visits for exhaustive territory mapping (Bibby et al., 2000), only two to three visits are conducted at each site (Knaus, 2018). Based on the records of three

(and above the timberline two) visits, the observers determine an estimate for the number of detected territories for each species and site according to established criteria (Mitschke et al., 2005; Schmid & Spiess, 2008; Sudfeldt et al. 2012). The estimated number of territories thus represents the aggregated result of all two to three visits in a given breeding season, forming the sole basis for population trend estimation for 78 regular breeding bird species (Strebel et al., 2024). Detection histories (Kéry, 2018) can be derived from these visits, consisting of two to three numbers that represent the number of territories found to be occupied during each respective visit. Herewith, we make the data resulting from the Swiss Common Breeding Bird Monitoring publicly available.

Class I. Data Set Descriptors

A. Data set identity:

Data of the Swiss Common Breeding Bird Monitoring Program

B. Data set identification codes:

header-species-count.csv

data-territories.csv

C. Data set description

Principal investigator: Hans Schmid, Swiss Ornithological Institute, Seerose 1, 6204 Sempach, Switzerland (Present: Klosterstrasse 5c, 6210 Sursee, Switzerland)

Abstract: The Swiss Common Breeding Bird Monitoring ("Monitoring Häufige Brutvögel", MHB) is a long-term study organized by the Swiss Ornithological Institute. Its main goal is to collect data for estimating breeding population trends. Since 1999, 267 one-kilometre squares laid out in a mostly systematic grid across Switzerland have been surveyed annually by skilled, mostly volunteer ornithologists. Bird populations are recorded using a simplified territory mapping protocol, with two visits per square above the timberline and three elsewhere. Surveys are conducted during the breeding season (mid-April to early July) along a square-specific transect route of four to six kilometers that does not change over the years. Each visit usually lasts three to four hours. The location of all visually or acoustically detected birds is recorded on a map or using a dedicated smartphone app. Records that meet predefined criteria in terms of species-specific breeding period and observed behavior are retained for the subsequent step of territory delimitation. This is done automatically for most species by the program Autoterri since 2022 and was done manually before, with a subsequent check by an expert. This process finally results in an estimate of the total number of detected territories per species, square and year. The design also explicitly generates detection histories, consisting of two to three numbers that represent the number of territories found to be occupied during each respective visit. The dataset currently covers the breeding seasons from 1999 to 2024 and includes 6,852 site-by-year combinations with estimates of detected territory numbers. It includes 162 of the 166 bird species recorded at least once as potential breeders, excluding four species to prevent potential disturbance at nesting sites. Besides informing about population trends, data from the Swiss Common Breeding Bird Monitoring have been used to illustrate several methodological developments, and to answer

scientific and applied questions. With its defined survey method, the systematic distribution of its survey sites, and the long timespan covered, it is likely that this data will continue to make important contributions in biological and biostatistical research. Herewith, we make the annually updated data set available with a CC-BY 4.0 license, allowing researchers and conservationists to use and analyze the data for their own research and conservation efforts.

D. Key words/phrases:
abundance, annual survey, breeding season, bird census, citizen science, common breeding bird monitoring, long-term monitoring, structured monitoring, Switzerland, territory mapping

Class II. Research origin descriptors

A. Overall project description:

1. Identity: Swiss Common Breeding Bird Monitoring, originally
2. Originators: Hans Schmid, Swiss Ornithological Institute, Seerose 1, 6204 Sempach, Switzerland (Present: Independent Researcher; Sursee, Switzerland)
3. Period of study: ongoing, starting in 1999
4. Objectives: The Swiss Common Breeding Bird Monitoring intends to monitor the populations of the more common species in terms of trends and changes in distribution.
5. Abstract: In 1999, the Swiss Ornithological Institute launched a monitoring program focusing on common and widespread breeding bird species in Switzerland. The distribution of the 267 annually visited one-kilometre squares is broadly representative of Switzerland up to 2500 m a.s.l. in terms of biogeographic regions, main habitats, and altitude. The surveys are carried out along a fixed route of typical length of four to six kilometers. The methodology corresponds to a simplified territory mapping method, with two visits per square above the timberline and three elsewhere. The location of all visually or acoustically detected birds is recorded on either a topographic map or using a dedicated smartphone app. Based on the two to three surveys, the territories are delimited. On average, about 34.1 species and a total of around 251 territories were detected per site and breeding season. A total of 166 species were recorded at least once as potential breeders in the Swiss Common Breeding Bird Monitoring program (Figure 1). For 78 of them, the Swiss Common

Breeding Bird Monitoring is the sole data basis for estimating their breeding population index in Switzerland; for another 39 species, the breeding population index is based on a combination of data from the Swiss Common Breeding Bird Monitoring and data from other sources like complete lists.

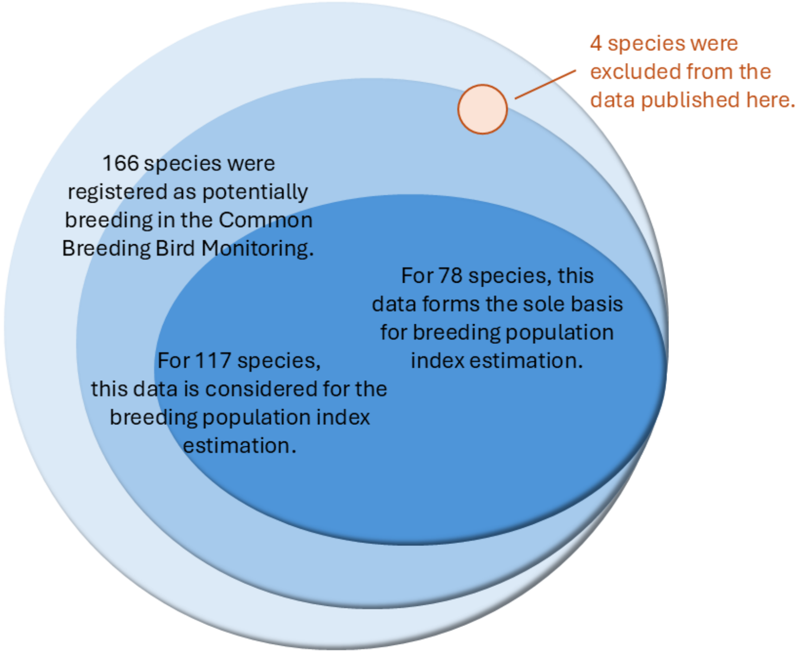

Figure 1. Schematic overview of species covered within the Swiss Common Breeding Bird Monitoring: Of the 166 species recorded at least once as potential breeders (i.e. meeting the inclusion criteria, see Class II, B3), data from this monitoring are used for breeding population index estimation for 117 species, serving as the sole basis for 78 of them. Four species are excluded from the data published here to prevent potential disturbance (see Class IV, C). Thus, data for 162 species is published.

6. Sources of funding:

   The Swiss Common Breeding Bird Monitoring is funded by the Swiss Ornithological Institute, a private and independent foundation supported by public contributions.

B. Specific subproject description

1. Site description:

   Switzerland is a landlocked country with a size of 41,285 square kilometers located in Western Europe. It consists of three main biogeographic regions, with the Alps in the south, the Jura mountains in the north-west and the Central Plateau in-between. The

country lies in the temperate zone, but climate can vary strongly as the country spans an altitudinal gradient from 193 to 4634 m a.s.l. In preparation for its 1999 launch, the sampling sites of the Swiss Common Breeding Bird Monitoring were selected from a grid defined for the Swiss Biodiversity Monitoring which comprises approximately 500 plots of one kilometer square each (Hintermann et al., 2000). Squares within that grid that either are at a median altitude above 2500 m a.s.l. or covered by at least 25% of lake surface were excluded. This led to a selection of 267 one-kilometer squares (Figure 2), among them 33 topographically challenging squares that were replaced by neighboring squares for safety reasons. The surface covered by the sample sites is largely representative for the country up to 2500 m a.s.l. in terms of biogeographic regions, habitat, and altitude (Schmid et al., 2004). The 267 one-kilometre squares are aligned with the Swiss grid (CH1903+ / LV95, EPSG:2056).

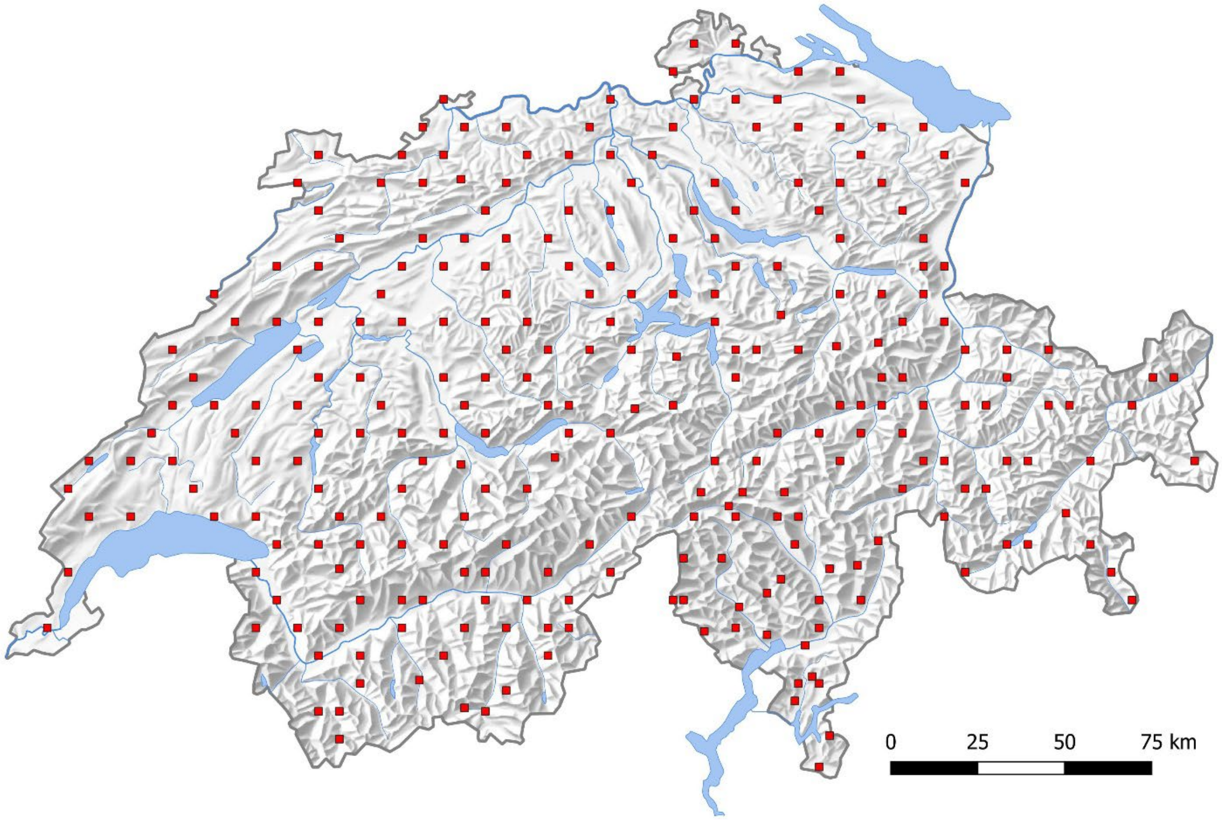

Figure 2. Location of the 267 one-kilometer sampling squares from the Swiss Common Breeding Bird Monitoring ("Monitoring Häufige Brutvögel" MHB). Sampling square symbols are not at scale to improve visibility. Base map: Swisstopo.

2. Sampling design

   Surveys are annually conducted during the breeding season along a predefined, square-specific transect route. Route adjustments are made only for safety reasons or impassability, typically in two to three cases per year. In such cases, the new route is matched in length and habitat composition to the original. A typical transect route has a length of four to six kilometers. It should provide a good overview of the square, covering all important habitats and elevation levels. Every year, all squares are visited three times below and twice above the timberline, respectively.

3. Research methods

   Bird populations in one-kilometer squares are monitored using simplified territory mapping, where instead of six to twelve repeat visits as recommended for exhaustive

surveys (Bibby et al., 2000), only two to three visits are conducted at each site (Knaus, 2018). This mapping method requires three (above the timberline two) visits per site, conducted during favorable conditions: early morning, wind not exceeding 3 on the Beaufort scale, and no rain. Before the breeding season, sites are allocated to observers, prioritizing those returning to their previous year's site. Surveys are conducted during the breeding season, between mid-April and beginning of June in the lowlands and starting at snowmelt in higher elevation sites. In each breeding season and for a given site, all repeat visits are typically conducted over a period of four to six weeks at the most. Surveys usually last about three to four hours and are conducted by one skilled ornithologist with binoculars. The location of all visually or acoustically detected birds or groups of birds is registered on a paper map or a tablet computer, including information on species identity (according to Gill & Rasmussen, n.d.), sex, age, behavior (singing, warning, flying over, transportation of nest material or food for chicks) or nest location. Counter-singing individuals of the same species indicating separate territories are depicted as such on a map or when entering the records into the app. This information has proven helpful for subsequent territory delimitation. The maps resulting from the two to three visits are subsequently combined for each species. Species by species, the observations meeting defined criteria in terms of breeding period and behavior are retained and grouped to territories. Each observation meeting these criteria needs to be assigned to a territory. Observations of simultaneously singing males help to correctly delimit the territories. Delimitation of territories is done by the program Autoterri (Wechsler, 2018 & 2020) since 2022 and was done manually before, with subsequent checks by an expert. This process results in an estimate for the number of found territories per species, site and year. The design also explicitly generates detection histories, consisting of two to three numbers that represent the number of territories found to be occupied during each respective visit. A territory typically contains one owner. For the sake of practicability, observers can aggregate multiple nests (e.g. at one building) or a group of lekking males into a single “territory”. For visits where such a territory was found to be occupied, the maximum number of either occupied nests or registered males

over all two to three visits in that "territory" goes into the sum of territories calculated for each visit.

Deducing detection histories is only possible if the exact locations of the detections are digitally available. Before 2013, only a fraction of mapping data is digitally available, thus only total territory numbers but no detection histories are available for these surveys.

4. Project personnel:

   Principal investigator: Hans Schmid

   Associated investigators: Samuel Wechsler, Guido Häfliger, Roman Bühler

   Technicians involved in data checking: Marcel Burkhardt, Christian Rogenmoser, Martin Spiess, Katarina Varga, Bernard Volet

   Observers: Between 1999 and 2024, >600 skilled field ornithologists participated in the field surveys.

Class III. Data set status and accessibility

A. Status

1. Latest update: January 2025
2. Latest archive date: August 2025
3. Metadata status: Last updated on August 2025, the version submitted.
4. Data verification: Observation data and resulting estimates for the number of found territories per species, site and year are checked and validated by technical staff after submission by the observers. From 2022 onwards, an automated data validation step is included, based on the comparison of several key figures like the total number of detected territories and species or the survey durations. Surveys (site-by-year combinations) assessed to be of insufficient quality are not retained. On average, this only affects a low single-digit number per year.

B. Accessibility

1. Storage location and medium: The data set is available as Supporting Information to this data paper in *Ecology* and is additionally available in the vogelwarte.ch Open Repository and Archive, hosted by Zenodo, in Strebel et al. (2025) at

https://doi.org/10.5281/zenodo.15854964. For the latest annual update, please see https://doi.org/10.5281/zenodo.15854963.

2. Contact person: Nicolas Strebel, nicolas.strebel@vogelwarte.ch
3. Copyright restrictions: none
4. Proprietary restrictions: CC-BY 4.0 license; cite this data paper when using the data
5. Costs: none

Class IV. Data structural descriptors

A. Data set file
   1. Identity:
      a. header-species-count.csv
      b. data-territories.csv
   2. Size:
      a. header-species-count.csv: 580 KB, 6852 rows
      b. data-territories.csv: 67.1 MB, 1,110,024 rows
   3. Format and storage mode: Comma-separated values (.csv)
   4. Header information: See variable information in section IV.B.
   5. Alphanumeric attributes: Mixed
   6. Special characters/fields: None
   7. Authentication procedures: None

B. Variable information

header-species-count.csv: Each row represents a survey, i.e. a combination of site and year. A survey consists of two to three visits, leading to an estimate of the number of territories per species. For the period of 1999 to 2024, this results in 6852 rows (267 sites × 26 years, with 90 surveys missing). The columns in *header-species-count.csv* are described in Table 1.

data-territories.csv: Each row represents an estimate of the number of found territories per species resulting from a survey (a combination of site and year). Until 2024, territories from 166 different bird species were registered in the data. Data from four among these 166 species are excluded from publication (see Class IV.C., data anomalies).

For the period of 1999 to 2024, this results in 1,110,024 rows (6852 surveys x 162 species). The columns in *data-territories.csv* are described in Table 2.

Table 1. Variable information in header-species-count.csv. Missing values are represented as NA.

**Variable name**	**Description**	**Potential values**
samplearea_id	Unique site identifier	Three-digit integer
x-koord, y-koord	Centroid coordinates of the one-kilometre square (CRS: CH1903+ / LV95, EPSG:2056)	Seven-digit integer
lon, lat	Centroid coordinates of the one-kilometre square (CRS: WGS 84, EPSG:4326)	Numeric in decimal degrees
year	Survey year	Four-digit integer
route_length	Length of transect route, in meters	Four-digit integer
median_elevation	Median elevation of the one-kilometre square, in m a.s.l.	Integer (range: 250–2750)
n_visits	Number of visits the survey consist of	Integer (2 or 3)
n_recorded_species	Number of species for which territories were registered at the survey	Integer (range by 2024: 3–64)

Table 2. Variable information in data-territories.csv. Missing values are represented as NA.

**Variable name**	**Description**	**Potential values**
species_id	Unique species identifier referring to the species code in the Swiss Ornithological Institute's database	Integer (range: 50 – 5740)
euring_id	EURING species code (euring.org/data-and-codes/euring-codes)	Integer (range: 70–18820)
nameeg	English species name (http://datazone.birdlife.org/species/taxonomy)	Character
namelt	Latin species name (http://datazone.birdlife.org/species/taxonomy)	Character
samplearea_id	Unique site identifier	Three-digit integer
year	Survey year	Four-digit integer
territories_visit_1, territories_visit_2, territories_visit_3	Number of territories found to be occupied per visit	Integer (range by 2024: 0–350)
territories_total	Number of found territories based on the two to three visits	Integer (range by 2024: 0–386)

C. Data anomalies:

In *header-species-count.csv*, 90 of the potentially 6942 (267 x 26) site-by-year combinations are missing. This is either because some sites were not surveyed every year, or that surveys were classified as being of insufficient quality. Together, this applies to 49 sites for one year, to eight sites for two years, to five sites for three years and to one site for ten years. *data-territories.csv* contains no data for these site-by-year combinations.

Data from the following species are excluded from *data-territories.csv* to avoid a potential risk for disturbance at nesting sites: Western Capercaillie *Tetrao urogallus*, Golden Eagle *Aquila chrysaetos*, Peregrine Falcon *Falco peregrinus*, Eurasian Eagle-Owl *Bubo bubo*.

The following patterns of missing values occur in *data-territories.csv*:

- territories_visit_3 missing: site above the timberline with only 2 visits (1218 surveys)
- territories_visit_1 to territories_visit_3 and territories_total missing: data for one or a few species in a survey not valid (191 records from a total of 122 surveys).
- territories_visit_1 to territories_visit_3 missing: Mapping data is not digitally available. In such cases it is not possible to deduce the detection histories. Only the total estimated number of territories is available (45,148 records from 1918 surveys, mainly from the first ten study years).
- territories_visit_1 or territories_visit_1 and territories_visit_2 missing: to leave aside migrants staging at a site, only observations from visits after a species-specific date limit are considered. However, if no territories resulted from the survey, territories_visit_1 to territories_visit_3 were set to 0 independent on whether the survey was conducted before or after the species-specific date limit.

Class V. Supplemental descriptors

A. Data acquisition

Field maps in A3-size are sent to the observers before the field season. In the field, observers note their observations on the maps. Afterwards they send the maps to the Swiss Ornithological Institute where they are scanned and stored. Once the field maps from all visits are scanned, the observer is notified to proceed with digitalization and

territory delimitation via the dedicated Web GIS at tmo.vogelwarte.ch. There, all locations of observed birds entered in the field maps must be digitized, including the information on sex, behavior and simultaneously registered observations. Since 2021, observations can be recorded digitally using a tablet or smartphone via a NaturaList App extension dedicated for Common Breeding Bird Monitoring, which transfers data directly to the Web GIS. Subsequently, records that meet certain criteria (in terms of species-specific breeding period, behavior and habitat) are grouped to territories by the observer (until 2021) or by the software Autoterri (from 2022 onwards). The observation data and the delimited territories are stored in the monitoring database of the Swiss Ornithological Institute. Records of subspecies are assigned to the corresponding species according to the regularly updated IOC World Bird List (Gill et al., n.d.).

B. Quality assurance/quality control procedures

After submission by the observers, the results of manual territory delimitation are checked and validated by technical staff (highly experienced ornithologists) according to a defined catalogue (Schmid & Spiess 2008). From 2022 onwards when territory delimitation is done by Autoterri, an automated data validation step is included, based on the comparison of several key figures like the total number of detected territories and species or the survey duration. Since the introduction of automated territory delimitation in 2022, the reviewing process is focusing primarily on checking and validating outliers. Based on the review, all surveys are classified as being of good, sufficient, or insufficient quality. Data from surveys classified as insufficient is not retained, affecting 0.7% of cases between 2013 and 2024, the period for which this information is available.

C. Related materials: none

D. Computer programs and data-processing algorithms:

1. Extension for common breeding bird monitoring data entry, embedded in the NaturaList App: NaturaList from Biolovision Sàrl company is a smartphone app linked to the online-portals of the ornitho family (e.g. ornitho.ch, ornitho.de). Both the app and the desktop versions allow entering observation data from several taxa. Since 2021, an extension specifically designed for collecting common breeding bird monitoring data is available, with a user interface mimicking data entry on a field map.

2. Autoterri: The program Autoterri was developed to delimit territories based on bird records of several seasonal visits conducted at a site. Comparison of automatic results with repeated manual delimitation by seven different experts showed very similar results with some variation between species (Wechsler 2018). Development and approach are described by Wechsler (2018 & 2020).

E. Archiving

The data set is available as Supporting Information to this data paper in *Ecology* and is additionally available in the vogelwarte.ch Open Repository and Archive hosted on Zenodo in Strebel et al. (2025) at https://doi.org/10.5281/zenodo.15854964. For the latest annual update, please see https://doi.org/10.5281/zenodo.15854963.

F. Publications and results:

Examples of publications using data from the Swiss Common Breeding Bird Monitoring can be found on https://www.vogelwarte.ch/en/projects/monitoring/monitoring-common-breeding-birds. The data was used to illustrate several examples in methodological textbooks, e.g. by Kéry & Royle (2016).

Acknowledgments

We are grateful to all observers that have participated in the monitoring program since its start in 1999. Patricia Dürring clarified important questions related to data license and archiving.

References

Baillie, S. R. (1991). Monitoring terrestrial breeding bird populations. In F. B. Goldsmith (Ed.), *Monitoring for Conservation and Ecology* (pp. 112–132). Chapman & Hall, London.

Bibby, C. J., Burgess, N. D., Hillis, D. M., Hill, D. A., & Mustoe, S. (2000). *Bird census techniques*. Elsevier.

European Environment Agency. (2021). Abundance and distribution of selected species in Europe (8th EAP). Retrieved from https://www.eea.europa.eu/ims/abundance-and-distribution-of-selected

Fraixedas, S., Lindén, A., Piha, M., Cabeza, M., Gregory, R., & Lehikoinen, A. (2020). A state-of-the-art review on birds as indicators of biodiversity: Advances, challenges, and future directions. *Ecological Indicators, 118*, 106728.

Gill, F., Donsker, D., & Rasmussen, P. (Eds.). (n.d.). *IOC World Bird List*. International Ornithologists' Union. Retrieved from https://www.worldbirdnames.org

Gregory, R. D., van Strien, A., Vorisek, P., Gmelig Meyling, A. W., Noble, D. G., Foppen, R. P. B., & Gibbons, D. W. (2005). Developing indicators for European birds. *Philosophical Transactions of the Royal Society of London B, 360*, 269–288.

Hintermann, U., Weber, D., & Zangger, A. (2000). Biodiversity monitoring in Switzerland. *Schriftenreihe Landschaftspflege und Naturschutz, 62*, 47–58.

Jiguet, F., Devictor, V., Julliard, R., & Couvet, D. (2012). French citizens monitoring ordinary birds provide tools for conservation and ecological sciences. *Acta Oecologica, 44*, 58–66.

Järvinen, O., & Väisänen, R. A. (1979). Changes in bird populations as criteria of environmental changes. *Ecography, 2*(2), 75–80.

Knaus, P. (2018). Territory mapping in kilometre squares. In P. Knaus, S. Antoniazza, S. Wechsler, J. Guélat, M. Kéry, N. Strebel, & T. Sattler (Eds.), *Swiss Breeding Bird Atlas 2013–2016: Distribution and population trends of birds in Switzerland and Liechtenstein* (pp. 64–65). Swiss Ornithological Institute, Sempach.

Kéry, M., & Royle, J. A. (2016). *Applied hierarchical modeling in ecology: Analysis of distribution, abundance and species richness in R and BUGS. Volume 1: Prelude and static models*. Academic Press.

Kéry, M. (2018). Identifiability in N-mixture models: A large-scale screening test with bird data. *Ecology, 99*(2), 281–288.

Mitschke, A., Sudfeldt, C., Heidrich-Riske, H., & Dröschmeister, R. (2005). Das neue Brutvogelmonitoring in der Normallandschaft Deutschlands – Untersuchungsgebiete, Erfassungsmethode und erste Ergebnisse. *Vogelwelt, 126*, 127–140.

PECBMS. (2023). Bird monitoring in Europe: countries. Pan-European Common Bird Monitoring Scheme. Retrieved May 2023, from https://pecbms.info/country/

Sauer, J. R., Link, W. A., Fallon, J. E., Pardieck, K. L., & Ziolkowski, D. J. (2013). The North American Breeding Bird Survey 1966–2011: Summary analysis and species accounts. *North American Fauna, 79*, 1–32.

Schmeller, D., Henle, K., Loyau, A., Besnard, A., & Henry, P. Y. (2012). Bird-monitoring in Europe – A first overview of practices, motivations and aims. *Nature Conservation, 2*, 41–57.

Schmid, H., Zbinden, N., & Keller, V. (2004). Überwachung der Bestandsentwicklung häufiger Brutvögel in der Schweiz. Schweizerische Vogelwarte, Sempach.

Schmid, H., & Spiess, M. (2008). Brutvogelaufnahmen bei BDM-Z7 und MHB: Anleitung zur Entscheidfindung bei Grenzfällen und zur Revierausscheidung. Schweizerische Vogelwarte, Sempach.

Sudfeldt, C., Dröschmeister, R., Wahl, J., Berlin, K., Gottschalk, T., Grüneberg, C., Mitschke, A., & Trautmann, S. (2012). Vogelmonitoring in Deutschland – Programme und Anwendungen. *Naturschutz und Biologische Vielfalt, 119*. Landwirtschaftsverlag, Münster.

Strebel, N., Antoniazza, S., Auchli, N., Birrer, S., Bühler, R., Sattler, T., Volet, B., Wechsler, S. & Moosmann, M. (2024). The State of Birds in Switzerland: Report 2024 www.vogelwarte.ch/state. Swiss Ornithological Institute, Sempach.

Strebel, N., Wechsler, S., Bühler, R., Häfliger, G., Keller, V., Kéry, M., Rogenmoser, C., Spiess, M., Varga, K., Volet, B., Zbinden, N., & Schmid, H. (2025). Supplementary material for

«Data of the Swiss Common Breeding Bird Monitoring Program» [Data set]. Zenodo. https://doi.org/10.5281/zenodo.15854964

van Turnhout, C. A. M., Hagemeijer, E. J. M., & Foppen, R. P. B. (2010). Long-term population developments in typical marshland birds in The Netherlands. *Ardea, 98*, 283–299.

Wechsler, S. (2018). Automating the analysis of territory mapping data in bird monitoring. Master's thesis, Paris Lodron University, Salzburg.

Wechsler, S. (2020). Autoterri – automatische Revierausscheidung für Brutvogelkartierungen in Terrimap Online. Methodenbeschrieb. Schweizerische Vogelwarte, Sempach.
